# Genetic diversity and phylogeny in *Hystrix* (Poaceae, Triticeae) and related genera inferred from Giemsa C-banded karyotypes

**DOI:** 10.1590/S1415-47572009005000053

**Published:** 2009-09-01

**Authors:** Hai-Qin Zhang, Rui-Wu Yang, Li Zhang, Chun-Bang Ding, Jian Zeng, Yong-Hong Zhou

**Affiliations:** 1 Triticeae Research Institute, Sichuan Agricultural University, SichuanChina; 2 Key Laboratory of Crop Genetic Resources and Improvement, Ministry of Education, Sichuan Agricultural University, SichuanChina; 3 College of Biology and Science, Sichuan Agricultural University, SichuanChina

**Keywords:** C-banding, *Elymus*, genome, *Hystrix*, *Leymus*, Triticeae

## Abstract

The phylogenetic relationships of 15 taxa from *Hystrix* and the related genera *Leymus* (**NsXm**), *Elymus* (**StH**), *Pseudoroegneria* (**St**), *Hordeum* (**H**), *Psathyrostachys* (**Ns**), and *Thinopyrum* (**E**) were examined by using the Giemsa C-banded karyotype. The *Hy. patula* C-banding pattern was similar to those of *Elymus* species, whereas C-banding patterns of the other *Hystrix* species were similar to those of *Leymus* species. The results suggest high genetic diversity within *Hystrix*, and support treating *Hy. patula* as *E. hystrix* L., and transferring *Hy. coreana*, *Hy. duthiei* ssp. *duthiei* and *Hy. duthiei* ssp. *longearistata* to the genus *Leymus*. On comparing C-banding patterns of *Elymus* species with their diploid ancestors (*Pseudoroegneria* and *Hordeum*), there are indications that certain chromosomal re-arrangements had previously occurred in the **St** and **H** genomes. Furthermore, a comparison of the C-banding patterns of the *Hystrix* and *Leymus* species with the potential diploid progenitors (*Psathyrostachys* and *Thinopyrum*) suggests that *Hy. coreana* and some *Leymus* species are closely related to the **Ns** genome of *Psathyrostachys*, whereas *Hy. duthiei* ssp. *duthiei*, *Hy. duthiei* ssp. *longearistata* and some of the *Leymus* species have a close relationship with the **E** genome. The results suggest a multiple origin of the polyploid genera *Hystrix* and *Leymus*.

## Introduction

*Hystrix* Moench is a small perennial genus of the tribe Triticeae (Poaceae). Moench (1794) established the genus *Hystrix* with *Hy. patula* Moench as the type-species through its distinctive morphological character of either lacking glumes entirely or, if present, of possessing long setaceous awn-shaped ones. Since then, 11 species have been included in *Hystrix* (Hitchcock, 1951; Bor, 1960; Tzvelev, 1976; Kuo, 1987; Osada, 1993). Baden *et al.* (1997) revised the genus and recognized six species, one of which is divided into three subspecies within *Hystrix*. All are tetraploids (2n = 4x = 28) except for *Hy. californica*, which is an octaploid (2n = 8x = 56). The natural distribution of *Hystrix* is disjunct with two species in North America (*Hy. patula* and *Hy. californica*), and the remainder in Central and Eastern Asia (Löve, 1984; Baden *et al.*, 1997).

Although separated early as a genus in its own right, the recognition of *Hystrix* has been controversial ever since its establishment. Church (1967a, 1967b) reported that *Hy. patula* had a close affinity to species of the *Elymus canadensis* complex and treated *Hy. patula* as *E. hystrix* L. Consequently, Dewey (1982) and Löve (1984) recognized the genus *Hystrix* as a section of *Elymus*. However, Jensen and Wang (1997) reported that two species of *Hystrix*, *Hy. coreana* and *Hy. californica*, shared the genome of *Leymus* (**NsXm**), and so transferred *Hy. coreana* from *Hystrix* to *Leymus*. Based on the results of studies on meiotic pairing and genomic *in situ* hybridization (GISH), Zhang *et al.* (2006) reported that *Hy. patula* possesses the *Elymus***StH** genome, whereas *Hy. duthiei* ssp. *duthiei* and *Hy. duthiei* ssp. *longearistata* contain the *Leymus***NsXm** genome. However, based on results of GISH and southern genomic hybridization, Ellenskog-Staam *et al.* (2007) reported that *Hy. coreana*, *Hy. longearistata* and *Hy. duthiei* contained the **Ns**^1^Ns^2^ genomes, while *Hy. patula* contained the **StH** genomes, and *Hy. komarovii* most likely had a variant of the **StH** genomes. Although the varied genomic constitution of *Hystrix* species has been reported, morphological similarities have likewise occurred, such as the loosely tufted caespitose, relatively high culms, broadly lanceolate leaves and the obsolete, reduced, or setaceous glumes. Thus, there is every indication that genome relationships and genetic diversity among *Hystrix* species need to be further investigated.

Karyotype analysis is considered to be an important method in genome analysis. Giemsa C-banded karyotyping stains constitutive heterochromatin, this resulting in unique banding patterns of individual chromosomes. This process thus provides more accurate evidence for identifying homologous chromosomes in karyologically similar species, thereby complementing studies of genome evolution among related species (Morris and Gill, 1987). Baden *et al.* (1997) undertook a pilot study on Giemsa C-banding patterns in *Hy. patula*, *Hy. komarovii* and *Hy. coreana*, and the results showed *Hy. coreana* as having large and conspicuous telomeric bands, different from the C-banding patterns of *Hy. patula* and *Hy. komarovii*. In this study, we investigated the genetic diversity among *Hystrix* species, as well as the phylogenetic relationship between *Hystrix* and its relatives (including related genera and their diploid ancestors) by using the Giemsa C-banded karyotype. The specific objectives were: (*a*) to report the Giemsa-C banded karyotypes of 15 perennial taxa in Triticeae representing nine genera; (*b*) to estimate genetic diversity among these perennial species; (*c*) to compare C-banding patterns among the diploid and tetraploid species; and (*d*) to explore the possible diploid ancestor of *Hystrix* species.

## Materials and Methods

###  Plant material

A total of 15 taxa in Triticeae were used in this study, including four taxa of *Hystrix* (2n = 4x = 28), three species of *Leymus* (2n = 4x = 28, **NsXm** genome), two species of *Elymus* (2n = 4x = 28, **StH** genome), two species of *Pseudoroegneria* (2n = 2x = 14, **St** genome), two species of *Psathyrostachys* (2n = 2x = 14, **Ns** genome), *Hordeum bogdanii* (2n = 2x = 14, **H** genome), and *Thinopyrum bessarabicum* (2n = 2x = 14, **E**^b^ genome) (Table 1). All seed material was collected in the field by the authors of this paper or kindly provided by the American National Plant Germplasm System (Pullman, Washington, USA) and Dr. S. Sakamoto (Kyoto University, Japan). Voucher specimens were deposited in the Herbarium of the Triticeae Research Institute, Sichuan Agricultural University, China (SAUTI).

###  Giemsa C-banding analysis

All seeds were germinated in Petri dishes on moistened filter paper at 22 °C. Root tips from the germinating seeds were pre-treated in ice-cold water for 24-28 h, fixed in ethanol: acetic acid (3:1, v/v) for 24 h at room temperature, and then stored in the refrigerator. Each root tip was squashed in a drop of 45% acetic acid.

The Giemsa C-banding technique followed the procedure of Gill *et al.* (1991). Metaphase cells with a complete chromosome complement were photographed, five cells being subsequently analyzed for each material. Idiograms so constructed were based on chromosome lengths, similarities in their morphology, banding patterns and relative arm-ratios. Chromosomes were arranged from the longest to the shortest and were designated with the Arabic numerals 1-7 in diploids and 1-14 in tetraploid species.

## Results

**Figure 1:**
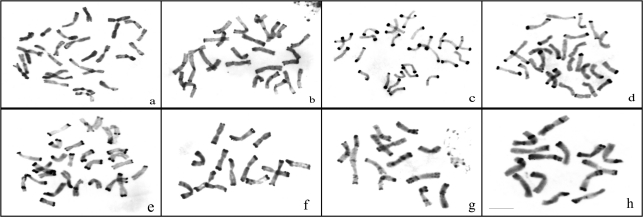
Giemsa-C banded metaphase chromosomes in representative species of *Hystrix* and relatives. **a.***Hystrix patula*. **b.***Hy. duthiei* ssp. *longearistata*. **c.***Hy. coreana*. **d.***Leymus arenarius.***e.***L. racemosus.***f.***Pseudoroegneria libanotica*. **g.***Hordeum bogdanii*. **h.***Psathyrostachys juncea.* Bar = 10 μm.

**Figure 2:**
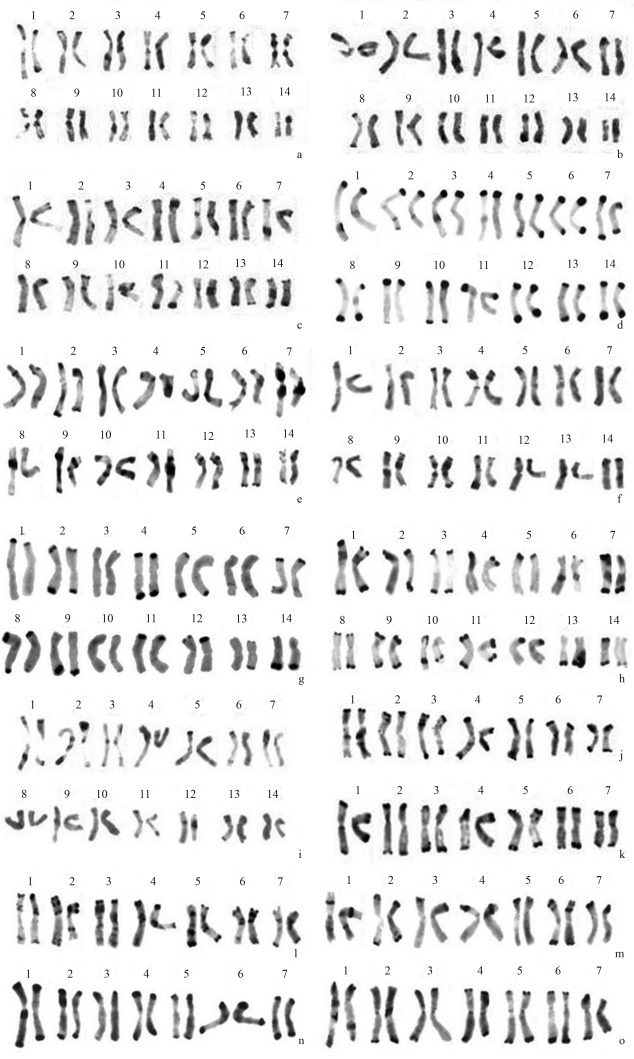
C-banded karyotypes in 15 taxa of *Hystrix, Elymus*, *Leymus,Pseudoroegneria*, *Hordeum*, *Psathyrostachys* and *Thinopyrum*. **a.***Hystrix patula*. **b**. *Hy. duthiei* ssp. *duthiei*. **c.***Hy.duthiei* ssp. *longearistata*. **d.***Hy. coreana*. **e.***Elymus sibiricus*. **f.***E. canadensis*. C-banded karyotypes in 15 taxa of *Hystrix, Elymus*, *Leymus,Pseudoroegneria*, *Hordeum*, *Psathyrostachys* and *Thinopyrum*. **g.***Leymus arenarius*. **h.***L. racemosus*. **i.***L. multicaulis*. **j.***Pseudoroegneria spicata*. **k.***Pse. libanotica*. **l.***Hordeum bogdanii*. **m.***Psathyrostachys juncea*. **n.***Psa. huashanica*. **o.***Thinopyrum bessarabicum*.

**Figure 3:**
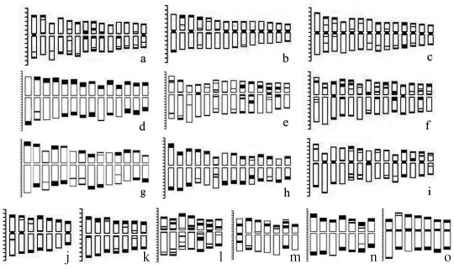
Ideograms in 15 taxa of *Hystrix, Elymus*, *Leymus,Pseudoroegneria*, *Hordeum*, *Psathyrostachys* and *Thinopyrum*. **a.***Hystrix patula*. **b.***Hy. duthiei* ssp. *duthiei*. **c.***Hy.duthiei* ssp. *longearistata*. **d.***Hy. coreana*. **e.***Elymus sibiricus*. **f.***E. canadensis*. **g.***Leymus arenarius*. **h.***L. racemosus*. **i.***L. multicaulis*. **j.***Pseudoroegneria spicata*. **k.***Pse. libanotica*. **l.***Hordeum bogdanii*. **m.***Psathyrostachys juncea*. **n.***Psa. huashanica*. **o.***Thinopyrum bessarabicum*.

The Giemsa-C banded metaphase chromosomes in representative species of *Hystrix* and relatives are shown in Figure 1. The C-banded karyotypes and ideograms of all the taxa in *Hystrix* and related genera are shown in Figures 2  and 3, respectively.

###  C-banding in *Hystrix* species

There were small telomeric bands in all of the 14 chromosomes in *Hy. patula* most of which with minor intercalary bands (Figure 1a). Large centromeric bands were also present in both arms of chromosomes 7, 8 and 9 (Figures 2a, 3a).

*Hy. duthiei* ssp. *duthiei* and *Hy. duthiei* ssp. *longearistata* revealed similar basic C-banding patterns. All the 14 chromosomes presented minor to small terminal and centromeric C-bands, except for chromosome 2 of *Hy. duthiei* ssp. *duthiei*, where centromeric bands were absent (Figures 2b, 2c). In *Hy. duthiei* ssp. *longearistata*, nine of the fourteen chromosomes contained minor interstitial bands, whereas these were present in only four of the 14 chromosomes in *Hy. duthiei* ssp. *duthiei* (Figures 3b, 3c).

Large terminal bands in all the 14 chromosomes were characteristic of the C-banding pattern in *Hy. coreana*. Except for a minor interstitial band in chromosome 2, no centromeric or interstitial bands were encountered in this species (Figures 1c, 3d).

###  C-banding in *Elymus* (StH), *Pseudoroegneria* (St) and *Hordeum* (H) species

Giemsa-C banded karyotypes in two *Elymus* species containing the **StH** genome were comprised of terminal, interstitial and a few centromeric bands. In *E. sibiricus*, small to medium terminal C-bands were observed in one or both arms of all the chromosomes, besides rather large interstitial bands in chromosomes 9-13 (Figures2e, 3e). In *E. canadensis*, all the chromosomes presented terminal C-bands in one or both arms. Furthermore, distinct bands were located near the centromere in both arms of chromosome 10, besides two pairs of chromosomes containing centromeric bands (Figures 2f, 3f). The banding pattern of *E. canadensis* is similar to that of *Hy. patula*.

In *Pseudoroegneriaspicata* (**St**) and *Pse. libanotica* (**St**), the C-banding patterns were rather similar, being characterized by large terminal bands in both arms or only in the short arm of all the seven chromosomes. Centromeric bands were found in chromosomes 1, 2 and 7 in *Pse. spicata* (Figures 2j, 3j), whereas in *Pse. libanotica* these were observed in chromosomes 1, 4, 6 and 7 (Figures 2k, 3k).

In *H. bogdanii* (**H**), small to medium interstitial bands were present in one or both arms of all the chromosomes, all of which showed terminal bands (Figures 2l, 3l).

###  C-banding in *Leymus* (NsXm), *Psathyrostachys* (Ns) and *Thinopyrum* (E) species

Although distinct terminal bands were present in the chromosomes of *L. arenarius* and *L. racemosus*, they were rather faint in those of *L. multicaulis*. All the 14 chromosomes of *L. arenarius* and *L. racemosus*, with the exception of chromosome 10 of *L. arenarius*, presented large terminal or interstitial C-bands and the absence of centromeric bands (Figures 2g, 3g; 2h, 3h). Small to medium terminal, interstitial and centromeric bands were found in the chromosomes of *L. multicaulis* (Figures 2i, 3i).

The C-banding patterns of the two *Psathyrostachys* (**Ns**) species were characterized by diagnostic terminal or interstitial bands in all the seven chromosomes (Figures 2m, n; 3m, n). One satellited chromosome (chromosome 6) and one chromosome with centromeric C-bands (chromosome 1) were observed in *Psa. juncea* (Figures 2m, 3m). Large terminal C-bands were observed in one or both arms of all the chromosomes in *Psa. huashanica*, although there were no centromeric bands (Figures 2n, 3n).

Distinct terminal and centromeric C-bands were noted in one or both arms of the seven *Th. bessarabicum* (**E**^b^) chromosomes. No interstitial bands were observed (Figures 2o, 3o).

## Discussion

###  Relationships among *Hystrix*, *Elymus* and *Leymus*

Cytological and molecular studies showed that species of *Hystrix* differed as to genomic constitution (Jensen and Wang, 1997; Mason-Gamer *et al.*, 2002; Zhang and Zhou, 2006; Zhang *et al.*, 2006; Ellenskog-Staam *et al.*, 2007; Fan *et al.*, 2007). *Hy. patula*, the type species of *Hystrix*, shared the **StH** genome of *Elymus*, whereas *Hy. coreana*, *Hy. duthiei* ssp. *duthiei*, *Hy. duthiei* ssp. *longearistata* and *Hy. californica* contained the **NsXm** genome of *Leymus*. In this study, the Giemsa C-banding patterns of four taxa of *Hystrix* were different. Furthermore, the C-banding patterns of *Hy. patula* were similar to those of *E. canadensis* and *E. sibiricus*. Darkly stained centromeric bands were observed in all the three species, although these were absent in the remaining *Hystrix* species. The results were consistent with those of chromosome pairing and GISH, hence suggesting a close relationship between *Hy. patula* and the *Elymus* species and a distant one between *Hy. patula* and the other species of *Hystrix*.

C-banding patterns of *Hy. duthiei* ssp. *duthiei* and *Hy. duthiei* ssp. *longearistata* were characterized by minor terminal and centromeric bands in almost all of the 14 chromosomes, thus displaying a certain degree of similarity with those of *L. multicaulis*. Nevertheless, there were differences in the number of minor interstitial bands. *Hy. duthiei* ssp. *longearistata* revealed 23 terminal bands, whereas *Hy. duthiei* ssp. *duthiei* only 16. Zhou *et al.* (1999) reported a certain morphological divergence and sterility barrier between the two taxa due to a difference in distribution and habitat. From previous cytological and molecular studies on our part, it was shown that the **NsXm** genomes of *Hy. duthiei* ssp. *duthiei* and *Hy. duthiei* ssp. *longearistata* were the same as those of the genus *Leymus* (Zhang *et al.*, 2006, 2008). In this study, the C-banding patterns of the two taxa were similar to those of *L. multicaulis*, although less centromeric and more interstitial bands were found in the latter. This indicated that *Hy. duthiei* ssp. *duthiei* and *Hy. duthiei* ssp. *longearistata* were closely related to *L. multicaulis*, which is congruent with results from cytological and molecular studies.

Jensen and Wang (1997) reported that *Hy. coreana* contained the **NsXm** of *Leymus* and so transferred the species to this genus. In this study, *Hy. coreana* revealed distinct terminal bands in all the 14 chromosomes, which similar to the banding patterns of *L. arenarius* and *L. racemosus*, and consistent with cytological and molecular studies.

###  Relationships between tetraploids and their diploid ancestors

From studies on chromosome pairing, there are indications that the **St** and **H** genome in *Elymus* originated from *Pseudoroegneria* and *Hordeum*, respectively (Dewey, 1967, 1971). In this study, C-banding diversity was observed among *Elymus* (including *Hy. patula*), *Pseudoroegneria* and *Hordeum*. Distinct terminal C-bands were observed, for example, in *Pseudoroegneria* (**St**), these being absent in tetraploid *Elymus* (**StH**) species. Similar results were found in *E. trachycaulus* (2n = 4x = 28, **StH**) and *Pse. spicata* (**St**) (Morris and Gill, 1987). These results suggested the occurrence of chromosomal re-arrangement between the **St** and **H** genomes in polyploidization events during the speciation process.

Previous cytological and molecular studies showed that species of *Leymus* have either **JN**, or **Ns**_1_Ns_2_, or **NsXm** genomes (Zhang and Dvorak, 1991; Wang *et al.*, 1994; Sun *et al.*, 1995; Anamthawat-Jónsson, 2005). The **J** (**E**) genome is from *Thinopyrum* and the **Ns** from *Psathyrostachys*. From this study it was indicated that two *Psathyrostachys* (**Ns**) species, besides *L. arenarius* (**NsXm**), *L. racemosus* (**NsXm**) and *Hy. coreana* (**NsXm**), had large terminal bands. However, the C-banding patterns of *Th. bessarabicum* (**E**^b^), *L. multicaulis* (**NsXm**), *Hy. duthiei* ssp. *duthiei* (**NsXm**) and *Hy. duthiei* ssp. *longearistata* (**NsXm**) were characterized by centromeric and terminal bands. From the results it could be inferred that *Hy. coreana* and some species of *Leymus* were closely related to the **Ns** genome of *Psathyrostachys*, whereas for *Hy. duthiei* ssp. *duthiei*, *Hy. duthiei* ssp. *longearistata* and a part of *Leymus* species this was so with the **E** genome. The present data are consistent with previous cytological and molecular data, thereby suggesting large genetic diversity within the genera *Hystrix* and *Leymus*, and the multiple-origin of the polyploid genera *Hystrix* and *Leymus* (Sun *et al.*, 1995; Anamthawat-Jónsson and Bödvarsdóttir, 2001; Yang *et al.*, 2006; Zhang and Zhou, 2006).

###  The use of C-banding in the phylogeny of Triticeae

Giemsa C-binding has been widely used in chromosome identification, genetic mapping and studies on genome evolution of Triticeae species, ever since it was first reported (*e.g.*, Morris and Gill, 1987; Gill *et al.*, 1991; Linde-Laursen and Baden, 1994). The basic C-banding patterns of *Pse. spicata*, *H. bogdanii*, *Psa. juncea*, *Psa. huashanica*, *L. racemosus*, *L. multicaulis*, *Hy. coreana*, and *Hy. patula,* as exposed in the present study, are consistent with the findings from previous studies on C-banding (Linde-Laursen and von Bothmer, 1986; Morris and Gill, 1987; Wei *et al.*, 1995; Baden *et al.*, 1997; Wang *et al.*, 1999; Ge *et al.*, 2004). These findings imply that the C-banding technique is relatively stable and repeatable. The C-banding analysis undertaken in this study revealed genetic diversity and phylogenetic relationships among species from nine genera in Triticeae, consistent with available data on chromosome pairing and molecular evidence. Thus, the Giemsa C-banding technique can be used as a supplementary method for analyzing the genomic constitution of wild species, especially the Triticeae.

## Figures and Tables

**Table 1:** - Species of *Hystrix* and other closely related genera used in Giemsa-C banding analysis.

Species	2n	Genome	Accession n.	Origin
*Pseudoroegneria libanotica* (Hack.) D. R. Dewey	14	**St**	PI 228391	Ardabil, Iran
*Pseudoroegneria spicata* (Pursh) Á. Löve	14	**St**	PI 232138	Montana, United States
*Hordeum bogdanii* Wilensky	14	**H**	Y 1508	Xinjiang, China
*Psathyrostachys huashanica* Keng ex Kuo	14	**Ns**	ZY 3157	Shanxi, China
*Psathyrostachys juncea* (Fisch.) Nevski	14	**Ns**	PI 430871	Former Soviet Union
*Thinopyrum bessarabicum* (Savul. & Rayss) Á. Löve	14	**E**^b^	PI 531711	Crimea, Ukraine
*Elymus sibiricus* L.	28	**StH**	ZY 3041	Sichuan, China
*Elymus canadensis* L.	28	**StH**	PI 236805	Canada
*Leymus arenarius* (L.) Hochst.	28	**NsXm**	PI 272126	Alma-Ata, Kazakhstan
*Leymus racemosus*	28	**NsXm**	PI 478832	Montana, United States
*Leymus multicaulis* (Kar. & Kir.) Tzvelev	28	**NsXm**	PI 440325	Dzhambul, Kazakhstan
*Hystrix patula* Moench	28	**StH**	PI 372546	Ottawa, Canada
*Hystrix coreana* (Honda) Ohwi	28	**NsXm**	W_6_ 14259	Vladivostack, Russian Federation
*Hystrix duthiei* (Stapf) Bor ssp. *duthiei*	28	**NsXm**	ZY 2004	Sichuan, China
*Hystrix duthiei* ssp. *longearistata* (Hack.) Baden, Fred. & Seberg	28	**NsXm**	ZY 2005	Tokyo, Japan
